# Vulvovaginal candidiasis and current perspectives: new risk factors and laboratory diagnosis by using MALDI TOF for identifying species in primary infection and recurrence

**DOI:** 10.1007/s10096-021-04199-1

**Published:** 2021-03-13

**Authors:** Lívia Custódio Pereira, Amabel Fernandes Correia, Zita Dinis Lopes da Silva, Ceres Nunes de Resende, Fabiana Brandão, Rosane Mansan Almeida, Yanna Karla de Medeiros Nóbrega

**Affiliations:** 1 Vulvar Pathology Clinic, Department of Gynecology, Brasilia University Hospital, University of Brasilia, Brasília, DF Brazil; 2 Medical Biology Management, Center of Parasitology and Mycology, Central Public Health Laboratory of the District Federal (LACEN-DF), Brasília, DF Brazil; 3 Microbiology and Immunology Clinical Laboratory, Department of Pharmacy, Faculty of Health Sciences, University of Brasilia, Darcy Ribeiro Campus, Brasília, DF 70900-910 Brazil

**Keywords:** Candidiasis, *Candida*, Non-*albicans*, Recurrence, Vaginal vulvovaginitis, Vaginal dysbiosis, Intestinal dysbiosis

## Abstract

Vulvovaginal candidiasis (VVC), considered the second cause of genital infection among women, has pathogenic mechanisms still to be elucidated and unknown risk factors. Prevalence studies with laboratory diagnosis (at first diagnosis and recurrence) are uncommon, especially using MALDI TOF, used in this clinical, epidemiological, and laboratory study for evaluating candidiasis, and identifying unknown risk factors. To obtain clinical and epidemiological data, patients were questioned, and there was material collection. Samples collected were identified by using phenotypic and presumptive methods and confirmed by MALDI TOF. This study analyzed 278 patients, divided into symptomatic (*n* = 173) and asymptomatic (*n* = 105) groups. Regarding the main candidiasis symptoms (discharge, itching, and burning), only 50.3% of patients described these concomitant symptoms, showing a positive predictive value of 67.8%. Regarding the risk factors investigated, there was a statistical correlation between candidiasis and dairy products, gut transit, contraceptive use, respiratory allergy, and panty liners, describing new risk factors related to intestinal and vaginal dysbiosis. After *Candida* species analysis and confirmation, the primary prevalence was 80.9% (*Candida albicans*), 15.2% (non-*albicans*), 1% (*Rhodotorula mucilaginosa*), and 1.9% (unidentified species). In recurrence, the prevalence was 66.7% (*C*. *albicans*) and 33.3% (non-*albicans*). The presence of symptoms has low positive predictive value for the diagnosis of candidiasis, even when considering the classic triad of symptoms. Laboratory identification of yeast species is essential for correct treatment, preventing the resistance to antifungals and the high recurrence. In addition, dairy products and bowel habits, both related to intestinal and vaginal dysbiosis, may be associated with VVC.

## Introduction

*Candida* spp. vulvovaginitis, or vulvovaginal candidiasis (VVC), considered the second cause of genital infection among women in menacme [1], occurs by excessive multiplication, favored by predisposing factors of yeast, found in the vaginal microbiota of women in reproductive phase [2, 3].

Despite the high prevalence and a large number of risk factors (RFs) associated with infection, the pathogenic VVC mechanisms and recurrent vulvovaginal candidiasis (VVCR) have not yet been fully elucidated [4]. *Candida* yeasts migrate from the lower gastrointestinal tract to the adjacent vestibule and vagina, a route similar to that taken by vaginal *Lactobacillus* species [5–7]. The vaginal colonization, as well as yeast growth and germination, is enhanced by an estrogen-rich environment, which occurs in menacme, combined oral contraceptive use, and hormone replacement therapy [6].

Besides the estrogen-rich environment, other risk factors contribute to VVC development, including genetic factors determining host susceptibility to infection, inflammatory response development, vaginal microbiota dysbiosis [3, 8], sexual activity, hygiene and clothing habits, diseases such as diabetes mellitus [4], and atopy [9–11].

Among VVCR risk factors listed, vaginal microbiota dysbiosis, defined as an imbalance in this microbiota, has been most recently described as directly associated with infection pathogenesis. Vaginal microbiota (VMB) plays an important role in preventing colonization by pathogens [12] and is affected by internal and external factors including hormonal changes (estrogen), menstruation, intestinal microbiota (near the rectum), intimate hygiene habits, sexual interaction (sharing with a partner), and contraceptive use (COC) [13].

In this context, studies have shown that *Lactobacillus* spp., in particular *L*. *crispatus*, *L*. *jensenii*, and *L*. *gasseri*, are predominant in healthy VMB [14], acting through different mechanisms, and they form an important defensive barrier against *Candida* spp. infection [15–20].

Concerning diagnosis, although the clinical presentation—characterized by discharge, intense itching, and burning, accompanied or not by vulvitis with edema and fissure presence—is frequent in VVC, these symptoms are not specific and may be associated to other clinical conditions, such as desquamative vaginitis, cytolytic vaginitis, aerobic and anaerobic vaginitis, trichomoniasis, gonorrhea, and allergic symptoms [21].

Fresh examination is based on direct microscopy, although an important diagnosis means fungal infections [22] have low sensitivity, varying from 40 to 70%, because the result is dependent on microscopist experience [6]. In recurrent cases, culture is recommended; however, despite allowing species identification, it has low sensitivity [6, 23].

More recently, with the molecular age advent, methods employing DNA for fungal identification have been used which are able to provide accurate and faster results, demonstrating > 90% sensitivity in identifying *Candida* species [24–27].

Another alternative is mass spectrometry, an analytical technique that consists of the atoms or molecule ionization, their separation according to their mass/charge ratio (*m*/*z*), and then their identification and quantification [28]. At present, one of the most widely used methods for biomolecule analysis is matrix-assisted laser desorption ionization (MALDI), followed by detection in a time-of-flight (TOF) analyzer, MALDI TOF [29, 30]. MALDI TOF has been recognized as a fast and reliable tool for accurately identifying yeasts (more than 90% species identification specificity) since the spectra generated at identification are unique signatures of each microorganism [31–33].

Using these more accurate diagnostic methods, it has been possible to evaluate species prevalence involved in VVC etiology. Although about 80 to 90% have *Candida albicans* isolated as the etiological agent [2, 34–38], non-*albicans* (NA) species, such as *Candida glabrata*, *Candida krusei*, *Candida parapsilosis*, and *Candida tropicalis*, are no longer considered commensal species to be understood as etiological VVC agents [3, 4, 39] which may be responsible for up to 30% of VVCR episodes [21, 40].

The more accurate determination of *Candida* species is particularly important in VVCR cases, whose prevalence demonstrated by some authors ranged from 5 to 10% [41–43]. A recent systematic review about overall VVCR prevalence has estimated that approximately 138 million women are affected annually, which corresponds to 3871 cases per 100,000 women/year and about 372 million women will be affected by life-long VVCR [44]. In this context, it is noteworthy that the recurrence prevalence obtained by prospective studies with laboratory diagnosis is unclear since most studies calculate the rates based on epidemiological questionnaires applied to patients, whose symptoms are self-described and can be confused with similar symptoms from another vaginitis.

Thus, facing a problem affecting women from different ethnicities worldwide, every effort should be made to study VVC and VVCR. This infection is very prevalent, and causes great discomfort, especially in the recurrent form, limiting the quality of life, affecting mental health and sexual life [45]. In this sense, this study aimed to identify the etiologic agent of infections as accurately as possible, characterizing new RFs associated with infection and evaluating recurrence with laboratory diagnosis.

## Materials and methods

### Study design and ethics

The sample was composed of 278 women attending gynecology outpatient clinics (Brasília, DF, Brazil). Of these, 105 were asymptomatic and 173 had at least one vulvovaginitis symptoms, such as discharge, itching, burning, initial dyspareunia, dysuria, or malodor.

This study was conducted according to ethical principles (Declaration of Helsinki). All subjects signed an informed consent agreement to participate in this study, which was previously approved by the Research Ethics Committee (Faculdade de Medicina, Universidade de Brasília (CEP-FM-UNB), protocol number 1,572,449).

### Epidemiological, clinical, and laboratorial screening

#### Epidemiological and clinical

At the consultation, the researcher conducted an interview with the patient and completed the symptom and risk factor questionnaire (Table 1), and during the physical examination realized by an experienced gynecologist, a vaginal discharge sample was collected, in a swab with Stuart transport medium, and was sent for laboratory identification.

**Table 1:** Symptom and risk factor questionnaire

**Questions**	**Response choices**
Demographics	Age
	Marital status
Presence at of least one symptom	Yes
	No
Number of similar episodes in the last 12 months	≤ 1
	≤ 3
	≥ 4
Symptoms (answers Yes or No)	≥ 4
Medication use in the last 30 days	Discharge, itching, burning, dyspareunia, foul odor, dysuria
	Antibiotic, antifungal, or other
**Antecedents and habits**	
Antecedent obstetric	Number of pregnancies, number of births and number of abortions
Contraceptive method	Combined hormone, other (IUD, condom, other)
Time with current sexual partner	≤ 6 months
	6 months to ≤ 1 year
	≥ 1 year
	NA
Number of sexual partners in the last 12 months	0
	1–2
	3–4
Medication use, diseases (answers Yes or No)	Diabetes, use of oral hypoglycemic drugs, use of corticosteroids or immunosuppressants
Personal habits (answers Yes or No)	Smoking, regular condom use, vaginal shower, daily panty protector
Milk ingestion and derivatives	< 1 portion per day
	≥ 1 portion per day
Gut transit	Normal and altered (constipation or diarrhea)

*IUD* intrauterine device, *NA* not applied

#### Laboratorial

##### Isolation and conservation of fungal isolates

Fungal isolates were obtained from cultures at 35 °C within 24 h in Sabouraud dextrose agar tubes with chloramphenicol (SDAC) (HiMedia, Mumbai, India). All isolated samples were stored at 50% aqueous glycerol solution and frozen in 2.0-mL centrifuge microtubes at −20 °C for recovery and subsequent control, if necessary, and sent for identification.

### Phenotypic identification of *Candida* species

For the phenotypic identification, the germ tube test (GTT) and the presumptive identification in a chromogenic medium (CM) were performed.

GTT was performed to differentiate *Candida albicans* (GTT positive) from non-*albicans Candida* species (GTT negative). Four pure fresh colonies subcultured on SDAC for 24 h at 35 °C were inoculated into 0.5 mL of human serum in test tubes (5 mL) and incubated at 35 °C for 3 h. Results were observed by an optical microscope (×400) using slide and coverslip preparations. Positive results were considered as isolates that presented elongated projections, called “Germ Tubes.” A reference American Type Culture Collection (ATCC) strain of *Candida albicans* (ATCC 90028) was employed as a positive control.

CM was used for presumptive identification of *Candida albicans*, *Candida tropicalis*, and *Candida krusei*. These *Candida* species are differentiated from the production of differently colored colonies due to reaction with the chromogenic substrates present in the medium. From subcultures on SDAC for 24 h at 35 °C, pure and fresh colonies were obtained and inoculated at CM with handle and then incubated for 24 to 48h. Plate reading was performed by visual means, where colony presence of specific color for each *Candida* species was observed. As a positive control, reference strains of *Candida albicans* ATCC 90028, *Candida tropicalis* ATCC 28707, and *Candida krusei* ATCC 34135 were used.

### Identification using matrix-assisted laser desorption ionization time-of-flight

Mass spectrometry technique (MALDI TOF) use, a chemotaxonomic method for phenotypic identification of fungi, allowed the confirmatory identification of *Candida* spp.

The methodological technique principle uses whole yeast cells, which have molecular biomarkers, such as peptides or ribosomal proteins that are detected in the spectra generated after equipment reading. These protein spectra are characteristic of each fungal species, and functioning as the fingerprint of each microbial species, which are then compared to spectra deposited in databases that allow identification by comparability [46, 47].

Spectra were analyzed using VITEK MS® Knowledge Base (BioMérieux, Marcy-l’Étoile, France) version 3.0. For comparing peaks formed with the characteristic patterns of the microorganism species, genus, or family were performed, resulting in its identification. Results were considered valid when the percentages of identification probability were equal to or higher than 90.0% [48].

### VVCR evaluation

During the study period, forty-one symptomatic patients were evaluated for recurrence, with the researcher reapplying the symptom and risk factor questionnaire and collecting another sample for laboratory evaluation.

### Statistical analysis

Statistical tests were performed using GraphPad Prism version 8.00 software for Windows (La Jolla, CA, USA). To analyze VVC risk factors, Fisher’s exact test was used to verify if there was a correlation between VVC and the presence of each risk factor surveyed. The data were considered statistically significant when *p* ≤ 0.0500. In calculating positive predictive value (PPV), equation PPV = (*a* / (*a* + *b*)) was used, where *a* refers to patients with positive laboratory results, here called true positives, and b, false positive results.

## Results

### Population characterization

This study included 278 women divided into symptomatic (62.2%) and asymptomatic (37.8%) groups. Regarding the positivity of laboratory tests, 53.2% of symptomatic women had positivity of laboratory tests; asymptomatic patients, 12.4% (Table 2).

**Table 2:** Group evaluation characterized by age, marital status and symptom presense

Category	Symptomatic		Asymptomatic		Total
*n*	173 (62.2%)		105 (37.8%)		278
	Negative laboratory diagnosis	Positive laboratory diagnosis	Negative laboratory diagnosis	Positive laboratory diagnosis	
*n* groups	81 (46.8%)	92 (53.2%)	92 (87.6%)	13 (12.4%)	
Age					
Media ± SD	38.5 ± 5.0	38.5 ± 4.2	38.5 ± 11.3	40 ± 0.0	37.4 ± 7.7
Age category					
19–25	13 (16.0%)	8 (8.7%)	8 (8.7%)	0	29 (10.3%)
26–40	46 (57.0%)	61 (66.3%)	51 (55.5%)	7 (53.8%)	168 (59.6%)
41–55	17 (21.0%)	19 (20.7%)	27 (29.3%)	5 (38.4%)	69 (24.5%)
> 55	5 (6.0%)	4 (4.3%)	6 (6.5%)	1 (7.8%)	16 (5.6%)
Marital status					
Married	46 (56.8%)	54 (58.7%)	62 (67.4%)	8 (61.5%)	170 (61.1%)
Single	35 (43.2%)	38 (41.3%)	30 (32.6%)	5 (38.5%)	108 (38.9%)
Total					278

*SD* standard deviation

The mean age was 37.4 ± 7.7 years, and the prevalent age group was 26 and 40 years old (59.6% of the total), followed by the age group of 41 to 55 years. The age group > 55 years was the one that included the smallest number (Table 2). Regarding marital status, patients declared themselves married (61.3%) or single (38.7%). Data from questionnaires (clinical data and risk factors) and laboratory tests are presented in Table 2.

### Symptom prevalence evaluation and laboratory diagnosis

**Fig. 1:**
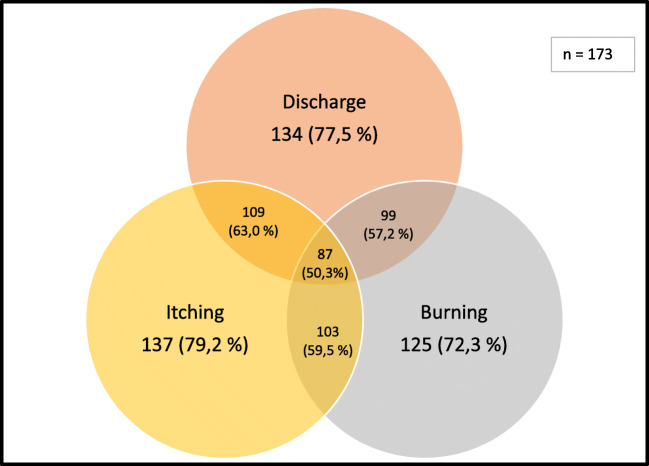
Venn diagram in symptomatic patients

Regarding clinical aspects evaluated in symptomatic patients, the most frequent symptoms were discharge, itching, and burning, and only 50.3% of the patients described these symptoms concomitantly (Fig. 1).

When associated with laboratory diagnosis, symptom presence had a positive predictive value (PPV) of less than 60% in individual analysis and in the presence of the three most frequent concomitant symptoms, PPV was 67.8% (Table 3).

**Table 3:** Symptom and laboratory diagnosis correlation

Category		Symptomatic		
*n*		173 (61.3%)		
	Total	Negative laboratory diagnosis	Positive laboratory diagnosis	PPV
*n* groups		81 (46.8%)	92 (53.2%)	
Discharge				
Yes	39 (22.5%)	24 (61.5%)	15 (38.5%)	57.5%
No	134 (77.5%)	57 (42.5%)	77 (57.5%)	
Itching				
Yes	36 (20.8%)	26 (72.2%)	10 (27.8%)	59.8%
No	137 (79.2%)	55 (40.1%)	82 (59.9%)	
Burning				
Yes	48 (27.7%)	30 (62.5%)	18 (37.5%)	59.2%
No	125 (72.3%)	51 (40.8%)	74 (59.2%)	
Presence of discharge, itching, and burning	87 (50.3%)	28 (32.2%)	59 (67.8%)	67.8%
Dyspareunia				
Yes	83 (48.0%)	38 (45.8%)	45 (54.2%)	54.2%
No	87 (50.3%)	41 (47.1%)	46 (52.9%)	
NA	3 (1.7%)	2 (66.6)	1 (33.4%)	
Bad odor				
Yes	155 (89.6%)	71 (45.8%)	84 (54.2%)	44.4%
No	18 (10.4%)	10 (55.5%)	8 (44.5%)	
Dysuria				
Yes	53 (30.6%)	19 (37.8%)	34 (64.2%)	64.2%
No	120 (69.4%)	62 (51.7%)	58 (48.3%)	

*PPV* positive predictive value

### Risk factor evaluation and laboratory diagnosis

**Fig. 2:**
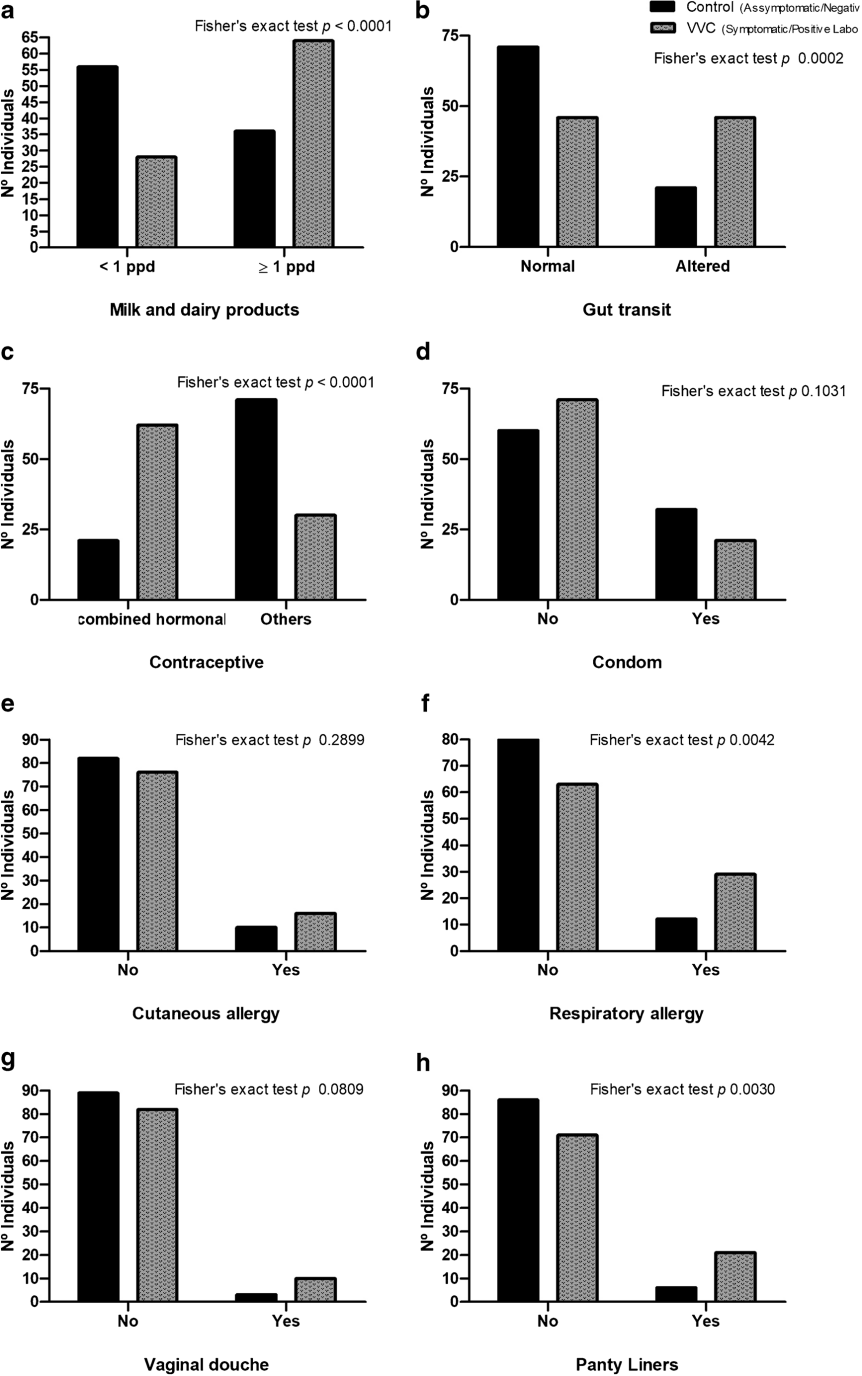
**a**–**h** Analyzed risk factors of vulvovaginal candidiasis. Symptomatic patients showing positive laboratory diagnosis (VVC group) were compared with asymptomatic patients, with negative laboratory tests (control group)

To analyze vulvovaginal candidiasis, risk factors were compared between symptomatic patients showing positive laboratory diagnosis (VVC group) and asymptomatic patients, with negative tests (control group) (Fig. 2).

Regarding the number of partners (last 12 months) and the time with the current partner, most patients in both groups had between one and two partners, and most women in both groups were with the same partner for more than one partner. No comparative analysis was performed for these two variables.

Patients in both groups were questioned about milk and dairy product consumption, which was categorized into less than one portion per day, or one or more portions per day. Comparative data analysis found that in the VVC group, ingestion was significantly higher when compared to the control group (*p* < 0.0001) (Fig. 2a).

Patients were also asked about their bowel habits, and chronic constipation or diarrhea was considered as altered with gut transit. In the VVC group, there was a significantly higher frequency of intestinal transit alterations when compared to controls (*p* 0.0002) (Fig. 2b).

About hormonal contraceptives, the usual frequency was significantly higher among the VVC group when compared to the control group, *p* < 0.0001 (Fig. 2c). Regarding condom use, there was no significant difference between the two groups, *p* 0.1031 (Fig. 2d).

Respiratory or skin allergies were also raised in both groups, and in relation to skin allergy, there was no significant difference between the two groups (*p* 0.2899) (Fig. 2e). However, in the case of respiratory allergy, the occurrence was significantly higher in the VVC group, with *p* < 0.0042 (Fig. 2f).

Regarding personal habits, there was no significant difference between groups concerning vaginal douche use (*p* = 0.2899) (Fig. 2g); however, among VVC groups, the frequency of daily panty liner use was significantly higher, *p* < 0.0042 (Fig. 2g).

### *Candida* species prevalence identified by laboratory diagnostic techniques

Species identification was performed by phenotypic means of the GTT and CM and was confirmed by MALDI TOF. Using MALDI TOF analyses, among 173 patients in the VVC group, 92 (53.2%) samples were positive for yeast, and among the 105 control group, 13 (12.4%) samples were positive (Table 4).

**Table 4:** Laboratory diagnosis by phenotypic methods and MALDI TOF

Laboratory diagnosis						
Symptomatic			Asymptomatic			Total
173 (62.2%)			105 (37.8%)			278
GTT	CM	MALDI TOF	GTT	CM	MALDI TOF	
75 positives (43.4%) 98 negatives (56.6%)	90 positives (52%) 82 negatives (47.4%)	92 positives (53.2%) 81 negatives (48.8%)	6 positives (5.7%) 99 negatives (94.3%)	13 positives (12.4%) 96 negatives (87.6%)	13 positives (12.4%) 96 negatives (87.6%)	
74 *C*. *albicans* (42.7%) 1 *Candida* spp. (0.6%)	79 *C*. *albicans* (48.4%) 12 non-*albicans* (7.4%)	78 *C*. *albicans* (45%) 13 non-*albicans* (7.5%) 1 other species (0.6%)	6 *C*. *albicans* (5.7%)	7 *C*. *albicans* (6.7%) 6 non-*albicans* (5.7%)	7 *C*. *albicans* (6.7%) 6 non-*albicans* (5.7%)	

*GTT* (germ tube test) is positive only for *C*. *albicans*, *CM* (chromogenic medium) differentiates yeast species from genus *Candida*

GTT, which is positive only for *C*. *albicans* species, was positive in 75 samples among the VVC group, while the chromogenic medium identified 79 *C*. *albicans*–positive samples (Table 4), suggesting that GTT failed to identify 4 *C*. *albicans* samples identified by CM and confirmed by MALDI TOF, or these strains have mutated and do not produce germ tube.

For the analyses performed in CM, which distinguishes *Candida* species, except for the failure to identify another species of yeast, all results were in agreement to MALDI TOF. MALDI TOF confirmed the diagnosis of 78 *C*. *albicans*, and identified a species among the VVC group unidentified by either GTT or CM, confirmed as *Rhodotorula mucilaginosa*.

The comparison between the results obtained by MALDI TOF (gold standard) and those obtained by GTT and CM made it possible to calculate the specificity and sensitivity of these laboratory methods compared to the gold standard (Table 5), when used in symptomatic and asymptomatic patients, giving a precise idea of the evaluation and limitations of these methods, which can be used when it is impossible to use MALDI TOF.

**Table 5:** Sensitivity and specificity of the methodologies employed in comparison to the MALDI TOF gold standard

Methodologies employed						
	GTT			CM		
	Symptomatic	Asymptomatic	Media	Symptomatic	Asymptomatic	Media
Sensitivity	84%	72%	78%	97%	93%	95%
Specificity	82%	97%	90%	98%	99%	99%

*GTT* germ tube test, *CM* chromogenic medium

**Fig. 3:**
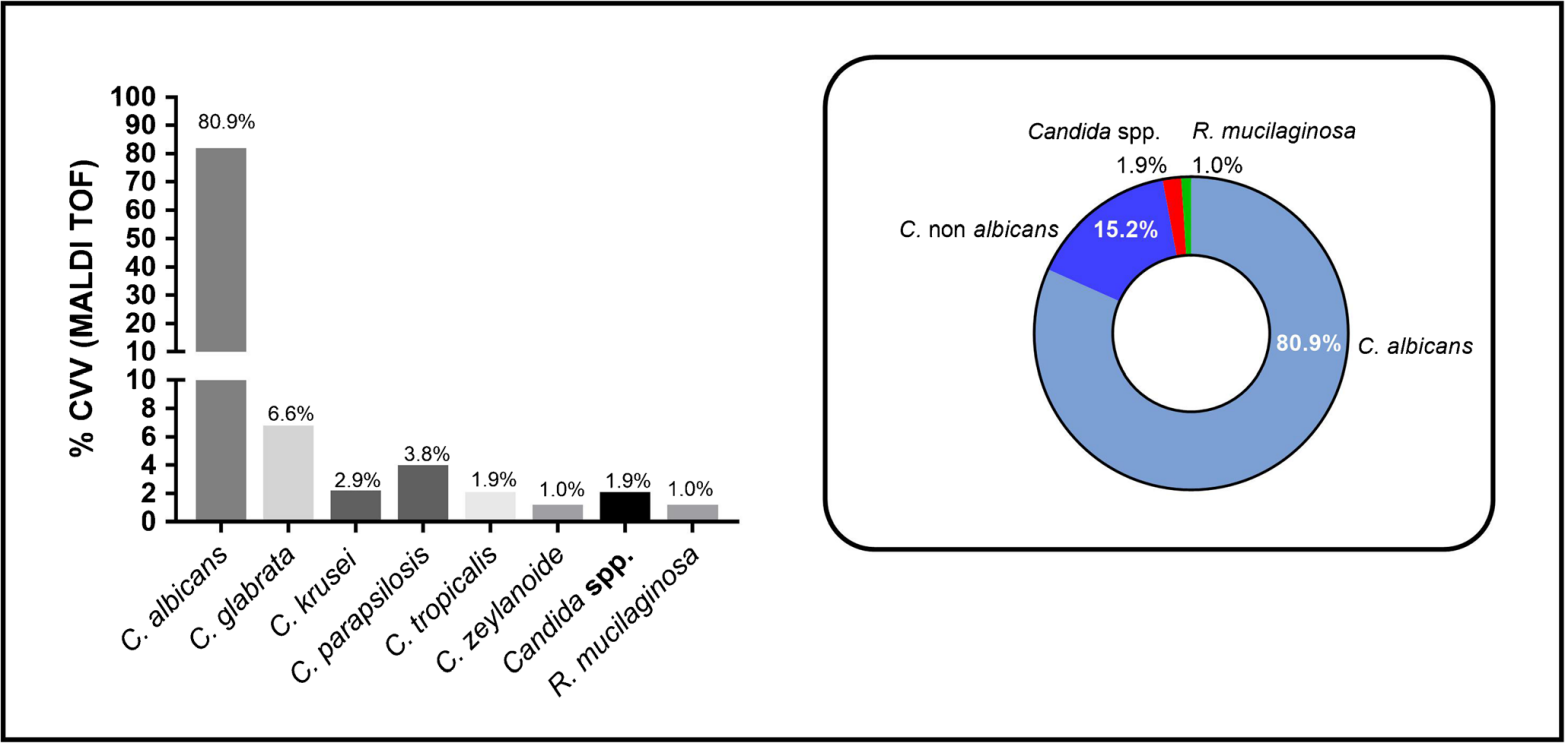
Identification of yeasts species by MALDI TOF

Sample analysis by MALDI TOF allowed the species identification from eight different yeasts, with a predominance of *C*. *albicans* (80.9%) followed by *C*. *glabrata* (6.6%), *C*. *parapsilosis*, *C*. *krusei*, *C*. *tropicalis*, and *C*. *zeylanoide* (Fig. 3). One *Candida* species was not identified and is described in this study as *Candida* spp. (1.9%) and another yeast, *Rhodotorula mucilaginosa* (1.0%), was identified. Therefore, the prevalence of non-*albicans* species (15.2%) from positive samples was identified (Fig. 3).

### Recurrence evaluation

**Fig. 4:**
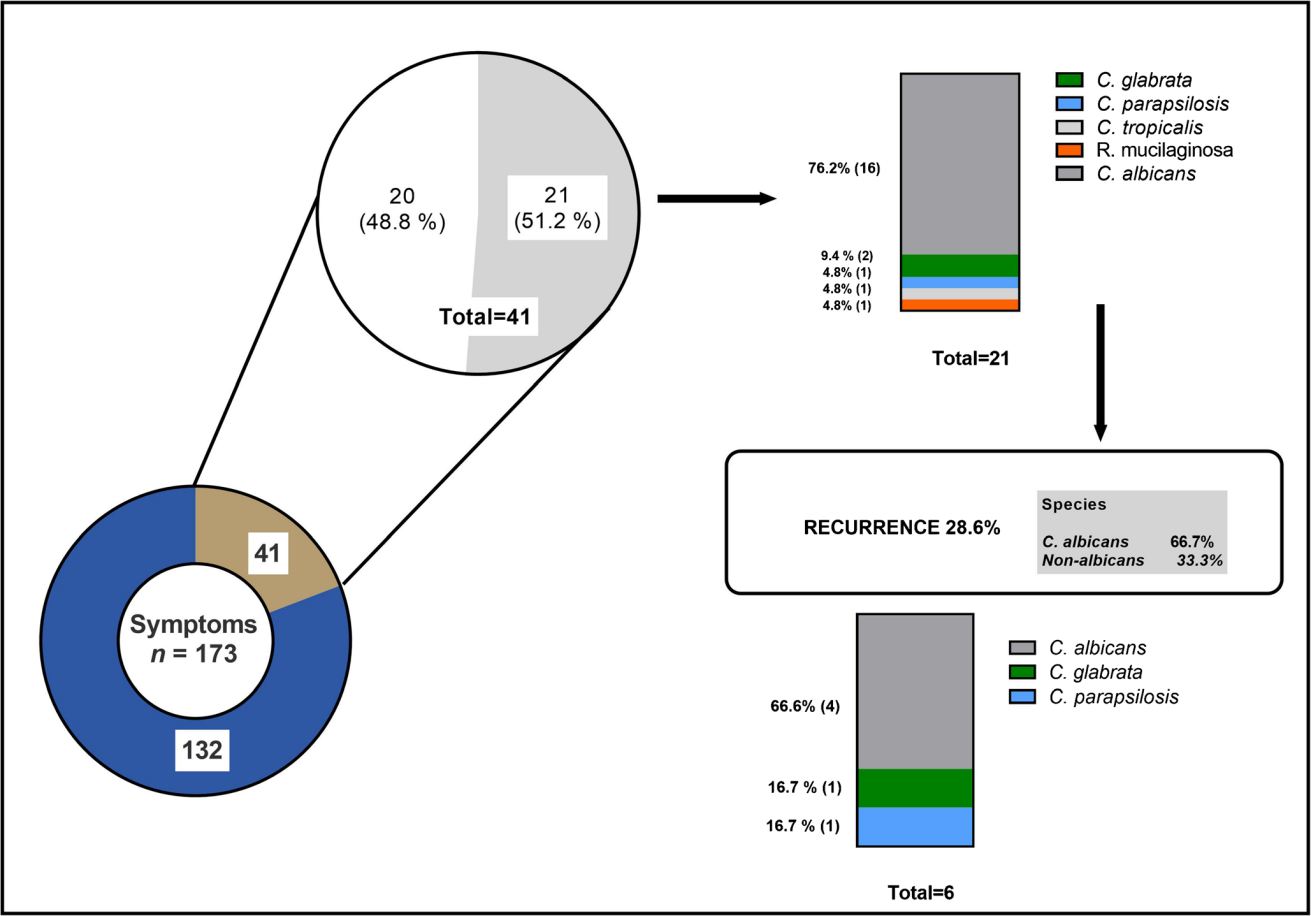
Laboratory assessment of RVVC in symptomatic patients

Among the 173 patients who were symptomatic during the first collection, 41 returned with symptoms and a second sample was collected (follow-up). Of these, 21 (51.2%) had positive MALDI TOF samples in the first collection (76.2% *C*. *albicans*, 23.8% other species). In the second collection, 6 (28.6%) of these 21 patients remained with positive laboratory diagnosis, showing 4 (66.7%) identified as *C*. *albicans* samples and two (33.3%) identified as non-*albicans* (Fig. 4).

## Discussion

The diagnosis of VVC is very often based on the presence of characteristic symptoms. These diagnoses are used not only in clinical practice but also in most epidemiological studies on the prevalence of the disease. However, some studies indicate that, due to the low specificity, diagnosis based only on the presence of symptoms is very imprecise [6, 49] and, in the case of a self-reported diagnosis, it can be wrong by up to 90% [33, 50].

Despite this data, in practice, the treatment of VVC in most cases is based on clinical diagnosis, and there are few studies that assess the predictive value of symptoms compared to laboratory diagnosis. In the present study, the laboratory diagnosis confirmed by MALDI TOF, a highly sensitive method, was compared to the symptoms reported by patients, and, only about half of the symptomatic patients had positive laboratory results. Even when the classic triad of symptoms was reported, the PPV of symptoms to diagnosis VVC was also low confirming that similar symptoms appear in vulvovaginitis to other etiologies, as well as in cytolytic vaginitis, aerobic vaginitis, trichomoniasis, vulvar dermatosis, and atrophic vaginitis [49, 51–57], which reinforces the need for laboratory diagnosis.

With respect to VVC-related risk factors, those frequently described in previous studies, such as oral contraceptive use, condoms, vaginal douche, and daily panty liners [4, 6, 58], were evaluated and, as well, those mentioned less frequently (respiratory and skin allergies) [10]. In addition to these risk factors already described, the study presented two new factors still little explored, namely the frequency of milk and dairy products, and changes in bowel habits. These factors were studied based on the current understanding that vaginal dysbiosis, defined as vaginal microbiota imbalance, may be directly implicated in the infection pathogenesis.

The laboratory method for identifying *Candida* spp. was MALDI TOF, with more than 90% specificity [32, 33, 59]. This method requires expensive equipment, not always accessible for diagnosis. The realization of classic laboratory methodologies such as GTT and CM, although currently uncommon, allowed an assessment of the sensitivity and specificity of each of them concerning the gold standard (MALDI TOF). This result makes it possible to carry out laboratory diagnostics in laboratories that do not have the MALDI TOF methodology available to use, which would justify the use of GTT and CM methodologies in the study, and would possibility the use of these in similar studies, with knowledge of the expected sensitivity and specificity in symptomatic and asymptomatic patients.

Another factor to be highlighted is that the CM in addition to permit the division into two distinct groups, the *albicans* species of the non-*albicans*, enables the identification of more than one species of *Candida* spp., when present in the same biological sample, which suggests the existence of VVC by more than one species, and does not represent contamination of the analyzed biological sample (data are not shown).

For many authors, *C*. *albicans* is considered a VMB and IMB commensal [51, 53, 60], and the onset of symptoms is attributed to a dysbiosis that results in excessive *Candida* growth [6, 11]. However, data on asymptomatic colonization are quite limited. A study in Australia using CM for culture found a prevalence of *Candida* spp. 21% [61], while in the present study, the prevalence was 12.4%.

This species is also the etiological agent of most cases of VVC [2, 33, 35–38, 62, 63]. Other authors pinpoint other species, such as *Candida glabrata*, *Candida krusei*, *Candida parapsilosis*, and *Candida tropicalis* as emerging [3, 4, 38]. In this study, the prevalence of *Candida albicans* (80.9%) and non-*albicans* (18.1%) was similar to previous studies. An unrelated yeast, *Rhodotorula mucilaginosa*, was also isolated. This species, considered non-virulent and saprophytic, was found in the genital region of 13.5% of asymptomatic people [64]. However, new studies show that it can be opportunistic [65, 66]. Another study identified this yeast in 10% of vaginal samples from pregnant women, 3.8% of these symptomatic [67]. Although the prevalence of this species was very low here, it confirms the existence of VVC caused by this species.

VVC recurrence data based on prospective studies, including laboratory analyses of samples from the same patient collected at different times, are uncommon. Most studies assess recurrence based on questionnaires, such as a study that includes data from several countries, which indicated a VVCR of 9% [44]. Another survey found a VVCR of 34% among women who answered a questionnaire [50]. In this study, the examination of the same patient who remained symptomatic, performed at different times, revealed a recurrence rate of 28.6%, and 33.3% for non-*albicans* species.

Some RFs that already described for VVC [4, 6, 10, 68] were evaluated. Confirming previous studies, the use of COC was greater when compared to controls. The use of a vaginal douche and condom did not differ between groups, perhaps due to the low prevalence of these habits.

The daily use of panty liners has been considered a risk factor for VVC [69, 70], but two reviews have not confirmed this association [71, 72]. In contrast, in this study, the use of panty liners was higher in the VVC group (*p* 0.0003). However, it cannot be affirmed that using panty liners is a risk factor, since women with VVC may be more likely to use them due to the presence of abundant secretion.

Some studies have linked the co-existence of VVC to respiratory allergies (RA), although they do not explain a possible mechanism [9, 73, 74]. The VVC group reported RA more frequently in the present study (*p* 0.0042). The immune response in individuals with RA is mediated by Th17, which increases IL-10, an anti-inflammatory cytokine that transfers the immune response (IR) to a Th2 pole [75]. Some components of the cell wall of glucan-rich fungi negatively modulate IR, displacing it to Th2, favoring the persistence of fungi, and reducing microbicidal effects on it [76]. Thus, an individual with RA has more IL-10, and this cytokine-mediated IR is ineffective in eliminating fungi like *Candida* spp., suggesting that this atopic individual would be more susceptible to VVC.

The study also presented two new RFs, frequency of ingestion of milk and dairy products, and changes in bowel habits. These factors were studied based on the current understanding that vaginal dysbiosis may be implicated in infection. VMB plays an important role in preventing colonization by pathogens and acts in maintaining women’s reproductive and gynecological health [12]. In addition, it is known that there is a correlation between IMB and VMB, including the terminal portion of the intestine as a source of colonization of the vagina by the migration of *Lactobacillus* spp. and yeasts [6, 51, 77].

There is a peak of lactase expression in the small intestine villi at birth, and this expression decreases with age, decreasing the absorption of lactose, which in contact with the IMB is fermented, producing H_2_, CO_2_ and methane (CH_4_), and short-chain fatty acids, which alter intestinal function [51]. Considering the most prevalent age group in the study, this deficiency in lactose absorption may result in an imbalance of the IMB, favoring the growth of yeasts, with interference in the VMB, given the correlation between the microbiome of the two sites.

Patients were asked about their bowel habits and classified by Rome IV consensus [71, 72]. In the VVC group, there was a higher frequency of changes in intestinal transit (*p* 0.0002). Studies show that, in individuals with intestinal constipation, bacteria of the genera *Bifidobacterium* and *Lactobacillus* decrease [48, 77–79]. Thus, the decrease of these species in the intestine, especially *Lactobacillus* spp., which have a recognized role in maintaining vaginal health, possibly reduces their presence in the vagina, since the VMB is fed-back by the IMB.

Our study makes new contributions to the diagnosis and clinic of VVC. Laboratory diagnosis of VVC is essential for treatment based on the identification of the *Candida* species, since non-*albicans* species are present in many of these infections and some of them have intrinsic or acquired resistance to azoles, and this therapy would be ineffective against these pathogens. In addition, there is a need for this diagnosis to rule out the presence of another non-*Candida* vaginitis, which share the same classic symptoms of VVC (discharge, itching, and burning), the most frequently cited in the clinic, and which in our study proved that they did present a low PPV in the diagnosis of VVC in these patients.

The existence of new risk factors associated with CVV, such as milk and dairy products and bowel habits, in addition to the use of panty liners and the presence of respiratory allergies, is relevant to the diagnosis of CVV, and may be related to the presence of dysbiosis of vaginal and/or intestinal. And the inclusion of these risk factors can add information to the clinical anamnesis, especially in patients who have the recurrent form of candidiasis (VVCR).

### Strengths and limitations of the study

Limitations in our study include the lack of a screening test, such as the Nugent criterion that could be performed using Gram stain, which would allow us to diagnose the presence of non-*Candida* vaginitis, in the absence and/or presence of CVV, making it possible to prove the existence of a single or mixed infection. Regarding the analysis of risk factors, the presence of a few patients using a vaginal shower and condom limited the analysis of these factors associated with CVV in the study.

The major strength of our study was to show the importance of laboratory diagnosis for the correct identification of CVV, and to compare it with the prevalence of the literature, which is almost always based on self-diagnosis in questionnaires answered by patients who describe the main symptoms of CVV. The study identified that the PPV based on the presence of symptoms is low when confirmed by the laboratory diagnosis, emphasizing the importance of the laboratory diagnosis for the clinical diagnosis. In addition, the description of new risk factors may contribute to the physician’s clinical conduct in conducting CVV treatment, namely the frequency of milk and dairy products, and changes in bowel habits, which may contribute to the understanding of vaginal dysbiosis.

## Conclusion

The presence of symptoms has low PPV for the diagnosis of candidiasis, even when considering the classic triad of symptoms. Laboratory identification of yeast species is essential for correct treatment, preventing the generation of resistance to antifungals, and the high prevalence of recurrent candidiasis as found in this study. In addition, new RFs, intake of dairy products, and bowel habits, both related to intestinal and vaginal dysbiosis, may be associated with VVC.
